# Age, sex, and mitochondrial-haplotype influence gut microbiome composition and metabolites in a genetically diverse rat model

**DOI:** 10.18632/aging.206211

**Published:** 2025-02-27

**Authors:** Hoang Van M. Nguyen, Eleana Cabello, David Dyer, Chloe Fender, Manuel Garcia-Jaramillo, Norman G. Hord, Steven Austad, Arlan Richardson, Archana Unnikrishnan

**Affiliations:** 1 Department of Nutritional Sciences, College of Allied Health, University of Oklahoma Health Sciences, Oklahoma City, OK 73117, USA; 2 Department of Microbiology and Immunology, College of Medicine, University of Oklahoma Health Sciences, Oklahoma City, OK 73117, USA; 3 Environmental and Molecular Toxicology, College of Agricultural Sciences, Oregon State University, Corvallis, OR 97331, USA; 4 Department of Nutritional Sciences, College of Education and Human Sciences, Oklahoma State University, Stillwater, OK 74075, USA; 5 Department of Biology, College of Arts and Sciences, University of Alabama at Birmingham, Birmingham, AL 35205, USA; 6 Department of Biochemistry and Physiology, College of Medicine, University of Oklahoma Health Sciences, Oklahoma City, OK 73104, USA; 7 Oklahoma Center for GeroScience and Healthy Brain Aging, University of Oklahoma Health Sciences, Oklahoma City, OK 73104, USA; 8 Oklahoma Veteran Affairs Medical Center, Oklahoma City, OK 73104, USA; 9 Harold Hamm Diabetes Center, OU Health, Oklahoma City, OK 73104, USA

**Keywords:** gut microbiome, aging, mitochondria, rat, metabolomics

## Abstract

We evaluated the impact of sex and mitochondrial-haplotype on the age-related changes in the fecal gut microbiome of the genetically heterogeneous rodent model, the OKC-HET^B/W^ rat. The age-related changes in the microbiome differed markedly between male and female rats. Five microbial species changed significantly with age in male rats compared to nine microbial species in female rats. Only three of these microbes changed with age in both male and female rats. The mitochondrial-haplotype of the rats also affected how aging altered the microbiome. Interestingly, most of the microbial species that changed significantly with age were mitochondrial-haplotype and sex specific, i.e., changing in one sex and not the other. We also discovered that sex and mitochondrial-haplotype significantly affected the age-related variations in content of fecal short-chain fatty acids and plasma metabolites that influence or are regulated by the microbiome, e.g., tryptophan derived metabolites and bile acids. This study demonstrates that the host’s sex plays a significant role in how the gut microbiome evolves with age, even within a genetically diverse background. Importantly, this is the first study to show that the mitochondrial-haplotype of a host impacts the age-related changes in the microbiome.

## INTRODUCTION

Since Lane et al. developed an efficient technique to sequence the 16S rRNA gene [1, 2], characterization of commensal microbes has illuminated the pleiotropic effects of the microbiome on health and disease. For example, studies have shown that the fecal gut microbiome (herein described as microbiome) influences the host’s metabolic health [3], immunity [4], cardiovascular health [5], and cognitive function [6, 7]. These effects in the host have drawn the attention of the aging community because dysbiosis, or perturbations in microbiome composition has been shown to increase with age and is believed to play a role in aging [8]. For example, age-related cognitive impairments such as Alzeimer’s [9, 10] and Parkinson’s diseases [11] have been associated with increases in the genera *Bifidobacterium* and *Akkermansia* respectively. A review by Badal et al. [12] shows that the human microbiome changes with age. However, there is little consensus on how age and the microbiome interact in humans because age-related changes in the host’s diet [13] and environment [14] can have a major impact on microbe abundance. These confounding variables make it difficult to draw conclusions on how aging specifically affects the microbiome in human studies. Use of laboratory rodents, where the diet and environment can be controlled throughout the life of an animal, allow investigators to directly test the impact of the aging host on its microbiome.

Studies in rodents support data in humans, which show that the microbiome changes with age in mice [15–18] and rats [19–21]. In addition, studies with mice have shown that aging interventions that increase lifespan and healthspan, such as caloric restriction or rapamycin, affect the age-related changes in the microbiome [22, 23]. However, several gaps in our knowledge must still be addressed to gain a better understanding of how the microbiome changes as the host ages. One problem is that many of the past studies in rodents only assess age-related changes of the microbiome in one sex in mice [15–18, 24] or rats [19–21]. Sex differences with age have also been largely ignored in human studies, with Badal et al. [12] reporting that 8 out of 27 papers did not disclose sex of their participants and 15 out of 27 studies combined male and female participants for statistical analysis. Data in humans and mice show that serum sex hormone levels can alter microbiome composition [25–27]. In addition, sex hormone levels decrease with age with the changes occurring differently in male and female humans and rodents [28–32]. Another limitation in the current studies with mice is that all have used inbred animals, which have negligible genetic variation. Because the genotype of the host can impact the microbiome [33–36], the lack of genetic variation in most rodent studies not only limits our knowledge of how aging affects the microbiome in other rodent genotypes but also limits the translation of the data from rodents to humans. In addition, previous studies in rodents have shown that the mitochondrial genome of the host affects the microbiome of young, inbred mice [37–39]. However, it is unknown if the mitochondrial genome can affect the gut microbiome in a model with nuclear heterogeneity, leaving a gap in knowledge which limits the translatability of these results to humans.

It is difficult to study the role of the mitochondrial genome in humans. Several factors contribute to this difficulty including recruiting humans with the exact same mitochondrial genome, herin termed mitochondrial-haplotype (mt-haplotype). A mother and her children and her sister and her children would have the same mitochondrial DNA sequence due to maternal inheritance of mitochondria, which limits the number of individuals who can be studied with identical mitochondrial genomes. Even then, these participants would be confounded by age and sex. Thus, most research studying the mitochondrial genome has relied on grouping people by mitochondrial-haplogroup (those with similar mitochondrial genome differences) or focusing on single nucleotide polymorphisms regardless of other differences in their mitochondrial genome. Thus, utilizing rodents allow researchers to study mt-haplotype of offspring from dams with the same mt-haplotype.

In this study, we have used a novel OKC-HET^B/W^ rat model to study the impact of age, sex, and mt-haplotype on the microbiome in a genetically heterogenous animal model. This rat model allows us to determine for the first time if mt-haplotypes, which have been shown to impact gut microbiome composition in young, inbred mice [37–39], has an impact on the age-related changes in the microbiome. We found that the abundance of various microbial species changed significantly with age in the genetically heterogenous OKC-HET^B/W^ rats. Importantly, most of the age-related changes in the microbiome were both sex and mt-haplotype dependent. In addition, we observed that the changes in the microbiome were associated with changes in short-chain fatty acids in the feces and in microbiome derived metabolites in the host’s plasma.

## RESULTS

### Changes in fecal gut microbiome composition with age differ by sex and mt-haplotype in OKC-HET^B/W^ rats

To examine how sex and mt-haplotype affect the age-related changes in gut microbiome composition, we used a rat model our group developed using a four-way cross strategy with four commercially available inbred rat strains (BN, F344, LEW, and WKY) as described in the Methods. Two F2 lines were created that were heterogenous with respect to the nuclear genome that had one of two mt-haplotypes: mitochondria from either BN (OKC-HET^B^) or WKY (OKC-HET^W^) rats. Rat body weights and composition are shown in Supplementary Figure 1. Male rats had increased body weight with age. While subcutaneous and gonadal fat tended to increase with age, the increase was not statistically significant. Female rats showed an increase in body weight and the weight of subcutaneous and gonadal fat with age. Interestingly, body weight and subcutaneous weight was significantly increased in the old female OKC-HET^W^ rats compared to the OKC-HET^B^ rats. In humans, specific mitochondrial haplogroups have been associated with obesity measured using BMI [40, 41]. Here, we show that mt-haplotype may affect specific fat pads in a sex-specific manner. After identifying commensal gut microbes in the feces based on the 16S rRNA sequence in these rats, our dataset contained 205 operational taxonomic units (OTUs). OTUs that appeared in only one sample were removed to reduce artifacts, leaving 149 features for the fecal samples from male and female rats.

**Figure 1:**
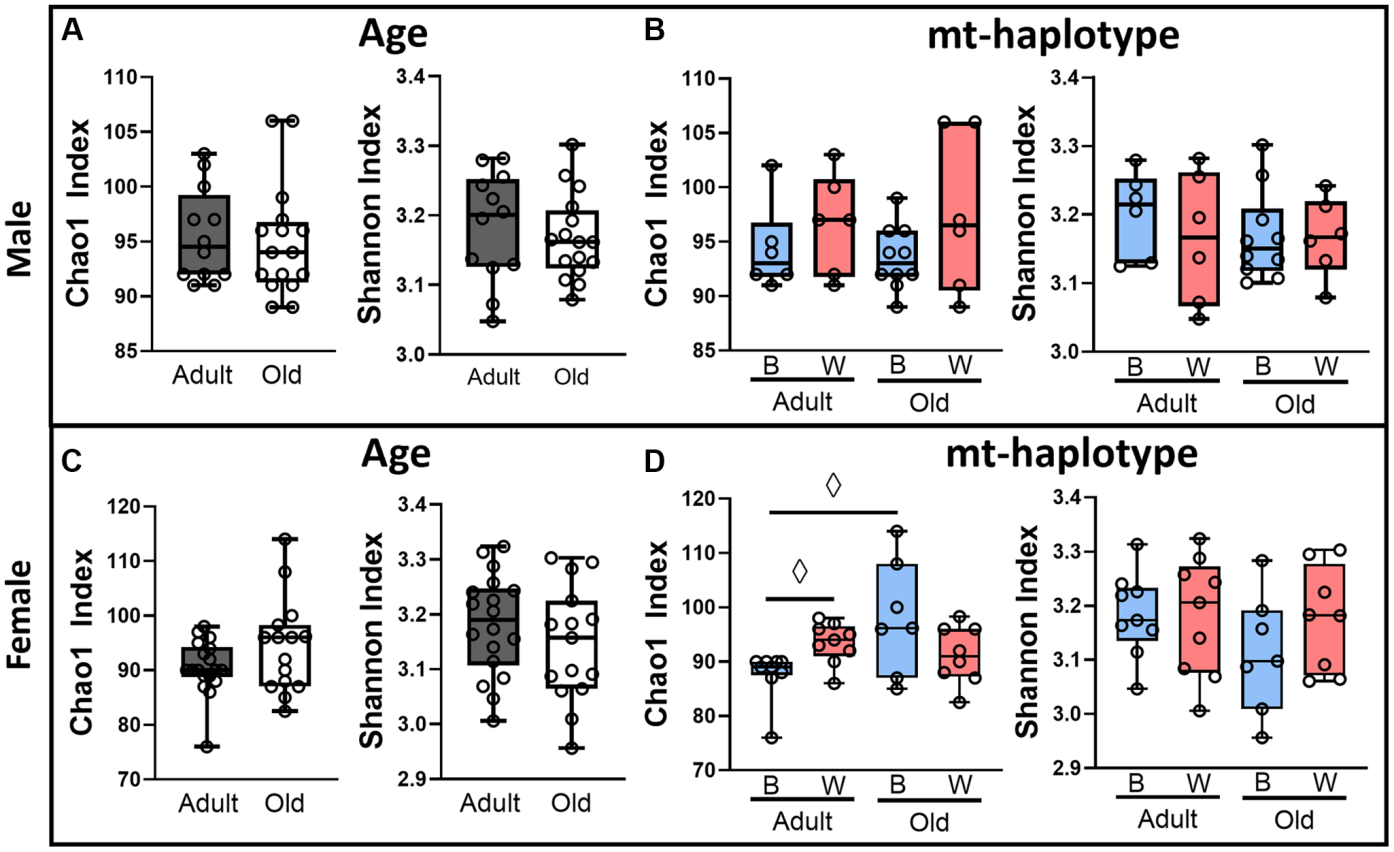
**Alpha-diversity is not significantly different with age and mt-haplotype in OKC-HET^B/W^ rats.** Alpha-diversity of the microbiome from adult (9-months) and old (26-months) OKC-HET^B/W^ rats was measured by the Chao1 and Shannon index. The alpha-diversity is shown for age (**A**) and mt-haplotype (**B**) comparisons for male rats; and age (**C**) and mt-haplotype (**D**) comparisons for female rats. Age comparisons were made by combining mt-haplotype groups for adult (gray boxes) and old (white boxes) animals. Mt-haplotype comparisons were made between OKC-HET^B^ (blue boxes) and OKC-HET^W^ (red boxes) groups. The data were collected from 6 to 10 rats per group, and the box plots display the 1st and 3rd quartiles with a horizontal line at the median. The whiskers display minimum and maximum values. ^◊^Values marginally significant by Fisher’s LSD *p* < 0.05.

We first measured alpha-diversity of the microbiome using both the Chao1 and Shannon Index to test for differences in the number of OTU’s (richness) and OTU abundance variance (evenness), respectively. There were no significant differences in either the Chao1 or Shannon Index when comparing male or female rats by age or mt-haplotype (Figure 1). However, the Chao1 index was marginally significant in adult female OKC-HET^B^ compared to the adult female OKC-HET^W^ and old female OKC-HET^B^ suggesting decreased richness in adult female OKC-HET^B^ rats.

**Figure 2:**
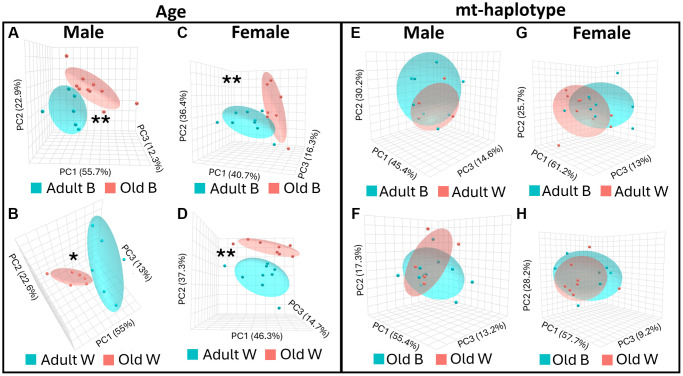
**Beta-diversity is different with age in OKC-HET^B/W^ rats.** Beta-diversity of the microbiome was measured using Jenssen-Shannon divergence comparing adult (9-months, blue ovals) and old (26-months, red ovals) rats for the following: male OKC-HET^B^ (**A**), male OKC-HET^W^ (**B**), female OKC-HET^B^ (**C**), and female OKC-HET^W^ (**D**) rats. Beta-diversity was also compared between OKC-HET^B^ (blue ovals) and OKC-HET^W^ (red ovals) haplotypes for the following: adult male (**E**), old male (**F**), adult female (**G**), and old female (**H**) rats. Oval outlines represent the 95% confidence interval for each group. The data were collected from 6 to 10 rats per group, and those values statistically significant by PERMANOVA at ^*^p < 0.05 or ^**^p < 0.01 are shown.

Next, we evaluated how the overall microbiome composition changed in male and female rats using beta-diversity; a dimension reduction technique to analyze differences in microbe populations between groups. Beta-diversity was visualized using principal coordinate analysis (PCoA). The PCoA plots in Figure 2 show age comparisons (adult vs. old) in rats of the same sex and mt-haplotype. In male rats, the microbiome composition was significantly different by age in both OKC-HET^B^ (Figure 2A) and OKC-HET^W^ rats (Figure 2B). A similar pattern was observed in female rats where beta-diversity was significantly different by age in OKC-HET^B^ (Figure 2C) and OKC-HET^W^ rats (Figure 2D). Differences in the microbiome composition by mt-haplotype (OKC-HET^B^ vs. OKC-HET^W^) was also evaluated using beta-diversity in rats of the same age and sex. There were no significant differences in beta-diversity when comparing mt-haplotype in either male or female rats (Figure 2E–2H).

**Figure 3:**
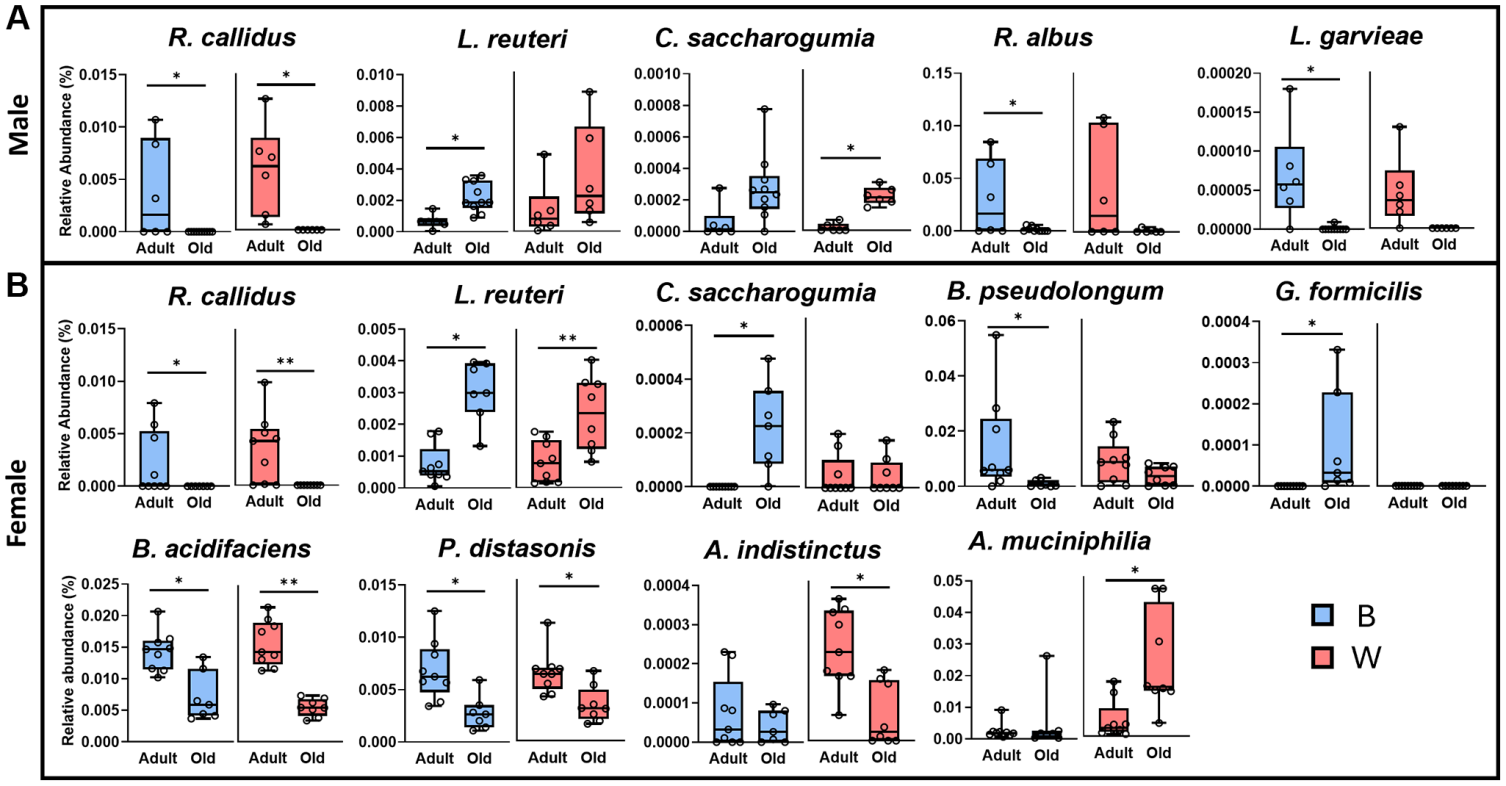
**Microbial species that are significantly changed with age are dependent on sex and mt-haplotype.** Microbial species in the gut microbiome from male (**A**) or female (**B**) rats were compared for adult (9-months) and old (26-months), OKC-HET^B^ (blue boxes) and OKC-HET^W^ (red boxes) rats. The box plots display the 1st and 3rd quartiles with the horizontal line denoting the median, and the whiskers display minimum and maximum values. The data were collected from 6 to 10 rats per group and statistically compared using Mann-Whitney/Kruskal-Wallis and/or Linear Modeling with significance at ^*^p < 0.05 or ^**^p < 0.01 shown.

Given the significant differences in beta-diversity by age, we next measured the abundance of microbial species that changed with age for male and female OKC-HET^B^ and OKC-HET^W^ rats. The identifiable species (45 species for male, 47 species for female) in our dataset accounted for 0.83–0.96% of the relative abundance for male, and 0.81–0.97% relative abundance in females. Most of the species that changed significantly with age were sex and mt-haplotype specific except for *R. callidus*, which decreased in both males and females (Figure 3). In males, the abundance of five microbial species were identified that changed significantly with age (Figure 3A). *R. callidus* abundance decreased with age in both mt-haplotypes. *L. reuteri* increased, *R. albus* decreased, and *L. garvieae* decreased in male OKC-HET^B^ rats but not OKC-HET^W^ rats. *C. saccharogumia* abundance was significantly increased in male OKC-HET^W^ but not in male OKC-HET^B^ rats.

In female OKC-HET^B^ and OKC-HET^W^ rats, we observed significant changes in the abundance of nine microbial species (Figure 3B), three of which also changed in male rats (*R. callidus, L. reuteri,* and* C. saccharogumia*). The abundance of *R. callidus, B. acidifaciens,* and *P. distasonis* decreased while *L. reuteri* increased with age in both female OKC-HET^B^ and female OKC-HET^W^ rats. In addition, the abundance of *C. saccharogumia* and* G. formicilis* increased while *B. pseudolongum* decreased with age in female OKC-HET^B^ but not female OKC-HET^W^ rats. In female OKC-HET^W^ rats, *A. indistinctus* abundance decreased and *A. muciniphilia* increased with age. In total, the abundance of four species of microbes changed significantly with age in male OKC-HET^B^ while only 2 species changed significantly with age in male OKC-HET^W^ rats. In female rats, the abundance of a similar number of microbe species changed in the two mt-haplotypes (e.g., 7 species for OKC-HET^B^ rats and 6 species for OKC-HET^W^ rats).

**Figure 4:**
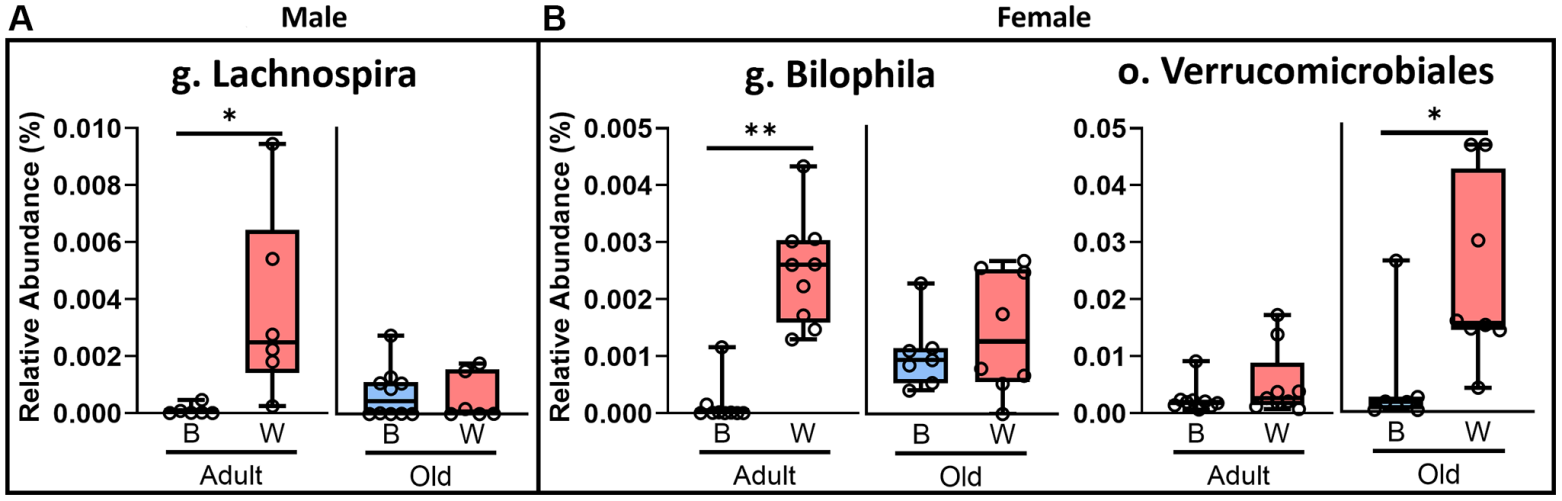
**The mt-haplotype affects several microbe groups in OKCHET^B/W^ rats.** Microbial OTUs are compared for OKC-HET^B^ (blue boxes) and OKC-HET^W^ (red boxes) male (**A**) and female (**B**) rats. The box plots display the 1st and 3rd quartiles with a horizontal line the median. The whiskers display minimum and maximum values. The data were collected from 6 to 10 rats per group, and the mt-haplotypes were statistically compared using Mann-Whitney/Kruskal-Wallis and/or Linear Modeling with significance at ^*^p < 0.05 or ^**^p < 0.01 shown.

We next compared the abundance of microbes at every level of taxonomy by mt-haplotype in rats of the same sex and age (Figure 4). We did not identify any significant differences at the species level. However, we observed that the abundance of genus *Lachnospira* was significantly increased in adult male OKC-HET^W^ rats compared to OKC-HET^B^ (Figure 4A) rats. In female rats, the abundance of genus *Bilophila* was significantly increased in the adult OKC-HET^W^ rats compared to adult OKC-HET^B^ rats (Figure 4B). In old female rats, a significant increase in the abundance of the order Verrucomicrobiales was observed in OKC-HET^W^ rats compared to OKC-HET^B^ rats (Figure 4B). In our dataset, the genera *Lachnospira* and *Bilophila* are individual OTUs and do not have species level data. *A. muciniphilia* is the only microbial species associated with the order Verrucomicrobiales in our dataset. Sparse Correlations for Compositional Data (SparCC) and Sparse Estimation of Correlations among Microbiomes (SECOM) algorithms were used to determine if microbial species correlated linearly with other microbial species in rats with the same age, sex, and mt-haplotype. We found no significant linear relationships between microbial species by age, sex, or mt-haplotype.

### Changes in fecal short-chain fatty acids (SCFAs) with age differ by sex in the OKC-HET^B/W^ rats

**Figure 5:**
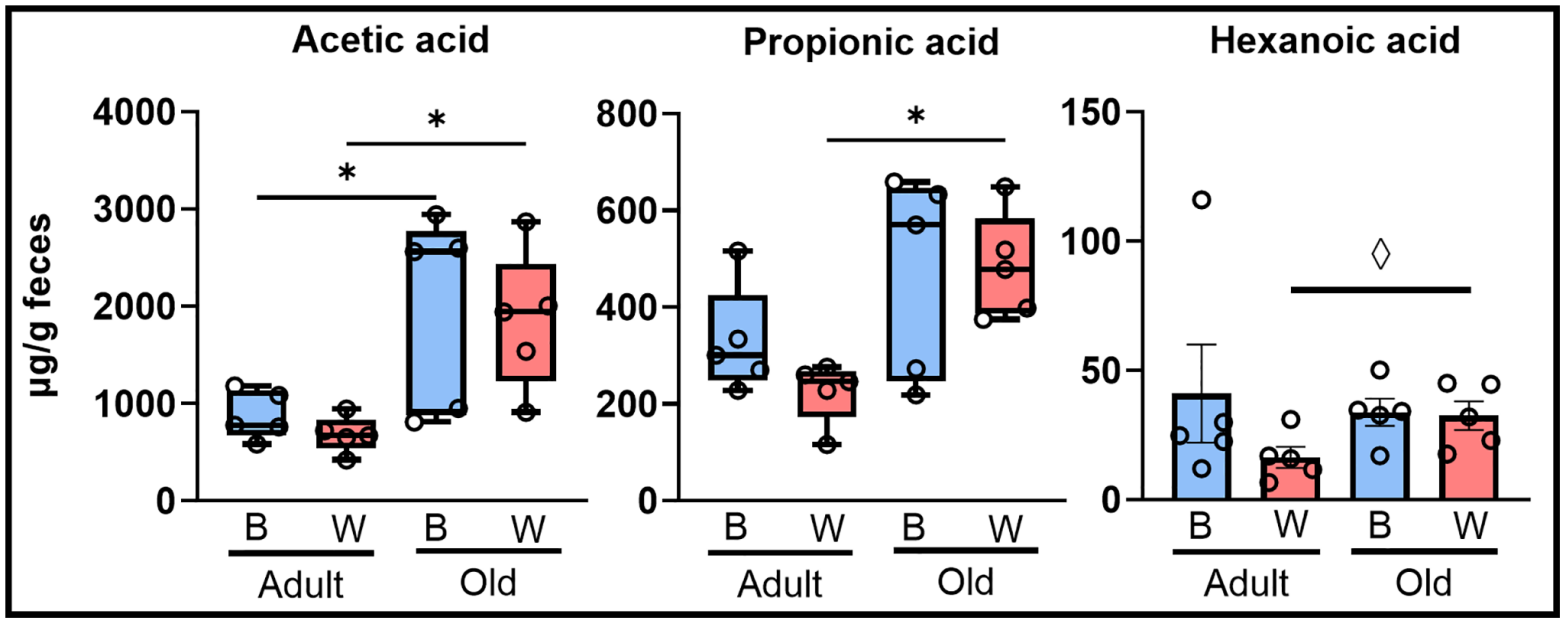
**Fecal short chain fatty acids changed with age in female rats.** Fecal SCFAs were measured in adult (9-months) and old (26-months), OKC-HET^B^ (blue boxes) and OKC-HET^W^ (red boxes) rats. Only female rats, shown here, were found to exhibit a significant difference with age in fecal SCFAs. The box plots display the 1st and 3rd quartiles with the horizontal line the median. The whiskers display minimum and maximum values. The data were collected from 5 rats per group, and values significantly different by FDR ^*^q < 0.05 or values marginally significant by Fisher’s LSD ^◊^*p* < 0.05 are shown.

SCFAs, such as butyric acid, acetic acid, and propionic acid, are generated from microbial fermentation of fiber. SCFAs have been shown to impact the host by regulating metabolic pathways in the host [42]. Therefore, we measured the abundance of SCFAs in the feces collected from the colon of our rats to determine if there were changes in SCFAs produced by the microbiome with age and mt-haplotype. Supplementary Figure 2A, 2B shows a heat map of the average normalized content of the eight SCFAs we detected in the fecal material. These heat maps of SCFAs profile suggested an increase in fecal SCFA in old OKC-HET^W^ rats regardless of sex. However, we only observed a significant age-related increase in total SCFAs in female OKC-HET^W^ rats and a marginal increase in female OKC-HET^B^ rats (Supplementary Figure 2C, 2D). Figure 5 shows the three SCFAs that changed significantly (FDR *q*-value < 0.05) or were marginally significant (Fisher’s LSD *p*-value < 0.05 and FDR *q*-value > 0.05) in female rats. In female rats, the increase in acetic acid was significant for both OKC-HET^B^ and OKC-HET^W^ rats when comparing age. Propionic acid levels increased with age in female OKC-HET^W^ rats, while the levels of hexanoic acid tended to increase with age in female OKC-HET^W^ rats. There were no significant changes in SCFAs when comparing mt-haplotypes in male or female rats.

### Changes in plasma tryptophan and bile acid metabolites with age differ by sex and mt-haplotype in the OKC-HET^B/W^ rats

Gut microbes are known to communicate with the host and influence health via microbe derived plasma metabolites such as tryptophan and bile acid metabolites [9, 43, 44]. Therefore, we measured the various tryptophan and bile acid metabolites in the plasma using an untargeted metabolomics dataset, which was obtained from the same rats that were used to study the microbiome. Dietary tryptophan is an essential amino acid that is metabolized by microbes into bioactive compounds. Supplementary Figure 3A–3F shows a heat map of the average levels of the nine tryptophan metabolites identified in the plasma isolated from OKC-HET^B^ and OKC-HET^W^ rats. Supplementary Figure 3G–3L show total abundance of different tryptophan derived metabolite classes. These metabolites arise from the three major arms of tryptophan metabolism: (1) indoles, (2) serotonin production, and the (3) kynurenine pathway. The most striking difference between the mt-haplotypes were observed in the indole profile of female rats which suggested that female OKC-HET^W^ rats had reduced plasma levels of the indole metabolites compared to the OKC-HET^B^ rats regardless of age. These data suggest that mt-haplotype led to changes in microbiome that led to changes in tryptophan derived indoles (Supplementary Figure 3D).

**Figure 6:**
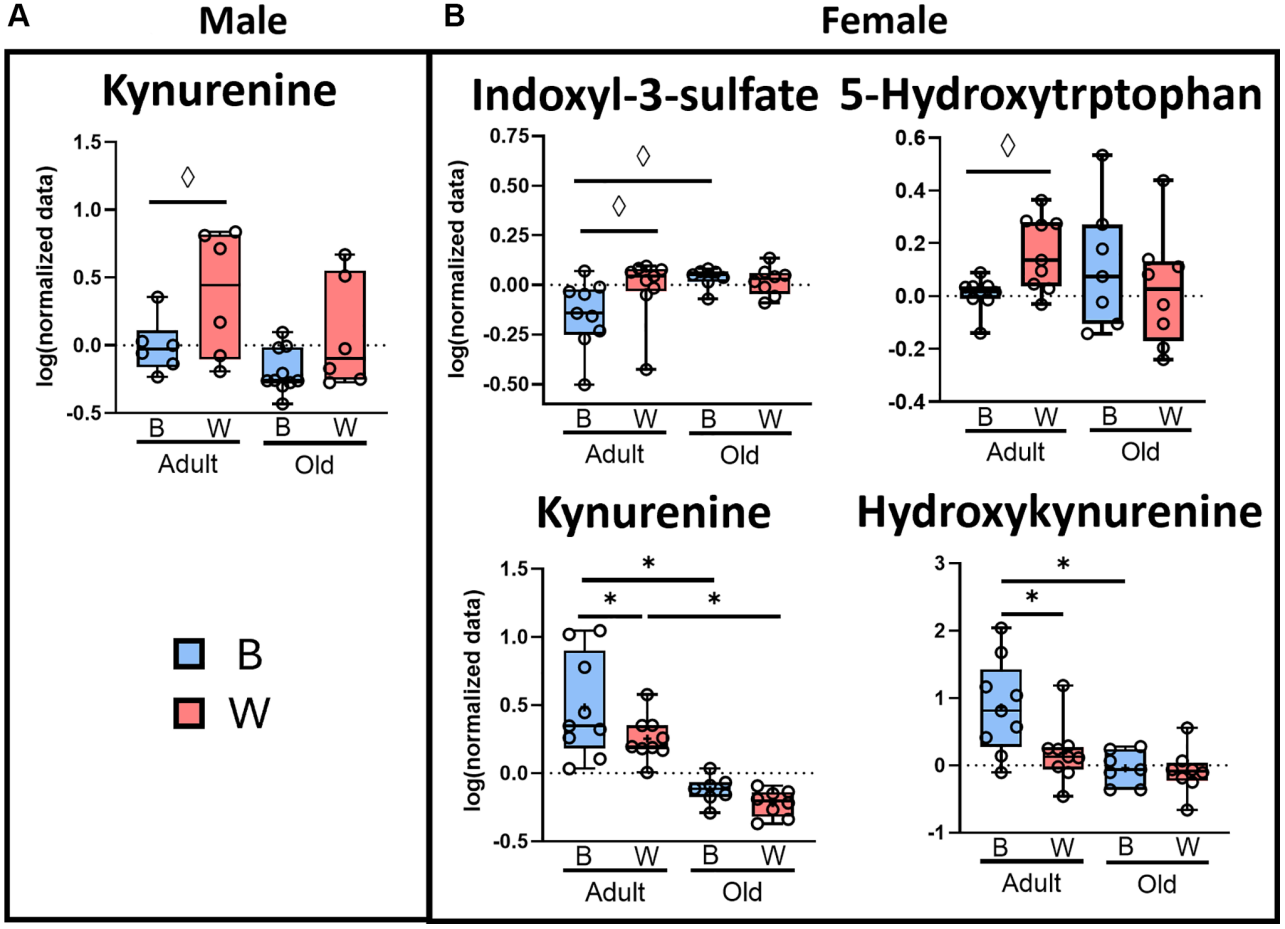
**Microbial metabolism of tryptophan to kynurenine may be affected by age and mt-haplotype in female rats.** Plasma tryptophan metabolites from male (**A**) and female (**B**) OKC-HET^B^ (blue boxes) and OKC-HET^W^ (red boxes) adult (9-months) or old (26-months) rats are shown. The box plots display the 1st and 3rd quartiles and a horizontal line at the median, and the whiskers display minimum and maximum values. The data were collected from 5 randomly selected rats per group, and the values significantly different by FDR ^*^q < 0.05 or values marginally significant by Fisher’s LSD ^◊^*p* < 0.05 are shown.

Figure 6 shows the levels of the plasma tryptophan metabolites that changed significantly (FDR *q*-value < 0.05) or were marginally significant (Fisher’s LSD *p*-value < 0.05) with age or mt-haplotype in male and female rats. In male rats, there were no tryptophan metabolites that changed significantly when comparing age or mt-haplotype; however, kynurenine was marginally increased in adult male OKC-HET^W^ rats compared to OKC-HET^B^ rats (Figure 6A). In contrast, female rats showed a significant change with age and mt-haplotype in several tryptophan metabolites (Figure 6B). Adult female OKC-HET^B^ rats had higher plasma content of kynurenine and hydroxykynurenine compared to old female OKC-HET^B^ rats. Additionally, indoxyl-3-sulfate levels were marginally increased with age in female OKC-HET^B^ rats. Only kynurenine levels decreased significantly with age in female OKC-HET^W^ rats. When comparing female rats by mt-haplotype, kynurenine and hydroxykynurenine plasma levels were significantly increased in the adult female OKC-HET^B^ compared to their OKC-HET^W^ counterparts, while the increase in 5-hydroxytryptophan levels were marginally significant in adult female OKC-HET^W^ compared to adult female OKC- HET^B^ rats. Interestingly, mt-haplotype differences only occurred in adult animals.

Bile acids are another mode of host-microbiome communication. Primary bile acids produced from cholesterol are conjugated with either taurine or glycine in the liver and released into the gastrointestinal tract in rats. Approximately, 95% of the bile acids are recycled through enterohepatic circulation. However, ~5% of the primary bile acids reach the colon and are metabolized by the gut microbiome to produce secondary bile acids and can be absorbed to facilitate host-microbiome communication [45].

**Figure 7:**
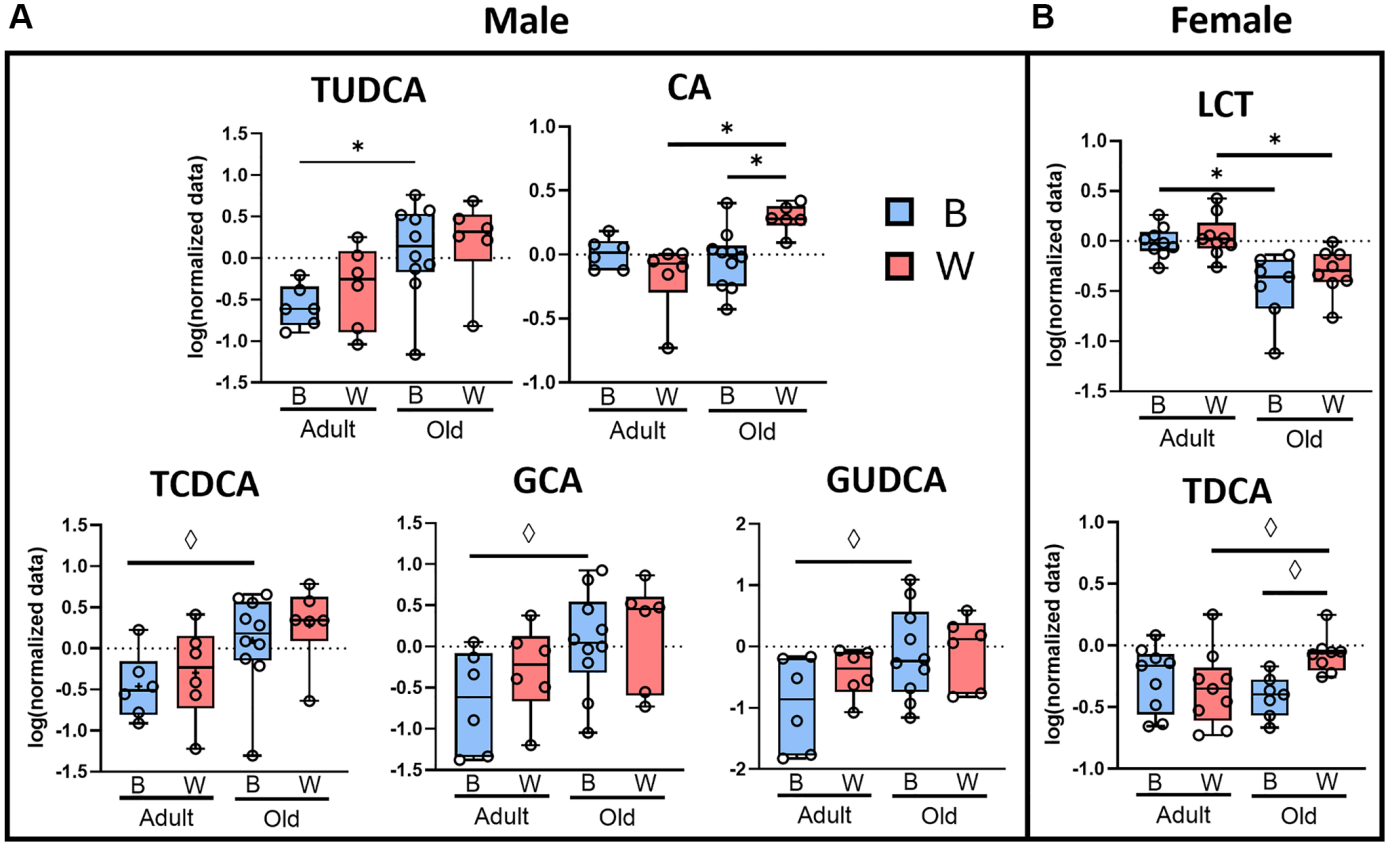
**Changes in primary, but not secondary bile acids, are dependent on sex and mt-haplotype.** Plasma bile acids from male (**A**) and female (**B**) OKC-HET^B^ (blue boxes) and OKC-HET^W^ (red boxes) adult (9-months) or old (26-months) rats are shown. The box plots display the 1st and 3rd quartiles with the horizontal line for the median, and the whiskers display minimum and maximum values. The data were collected from 6 to 10 rats per group, and significance was defined as FDR ^*^q < 0.05 or values marginally significant by Fisher’s LSD ^◊^*p* < 0.05. Abbreviations: TUDCA: tauroursodeoxycholic acid; CA: cholic acid; TCDCA: taurochenodeoxycholic acid; GCA: glycocholic acid; GUDCA: glycoursodeoxycholic acid; LCT: lithocholytaurine; TDCA: taurodeoxycholic acid.

Supplementary Figure 4 shows a heat map of the average plasma content of the fourteen bile acids and taurine that were detected in the plasma of the rats. In male rats, the overall bile acid profile suggests a robust increase in primary bile acids in plasma with age in both male OKC-HET^B^ and OKC-HET^W^ animals that was not apparent in female rats. Figure 7 shows the plasma content of the seven bile acids that changed with age and/or mt-haplotype. In male rats (Figure 7A), there was a significant increase in the levels of tauroursodeoxycholic acid (TUDCA) while the levels of taurochenodeoxycholic acid (TCDCA), glycocholic acid (GCA), and glycoursodeoxycholic acid (GUDCA) were marginally significant in male OKC-HET^B^ rats with age but not in OKC-HET^W^ rats. Cholic acid (CA) was the only bile acid that significantly increased with age in male OKC-HET^W^ rats. When comparing mt-haplotype in male rats, cholic acid levels were significantly increased in old male OKC-HET^W^ rats compared to their OKC-HET^B^ counterparts. In female rats (Figure 7B), the change in bile acids was limited to two metabolites with only plasma levels of lithocholytaurine (LCT) showing a significant decrease with age in both OKC-HET^B^ rats and OKC-HET^W^ rats (Figure 7B). The levels of taurodeoxycholic acid (TDCA) levels were marginally increased in old female OKC-HET^W^ rats compared to adult OKC-HET^W^ rats and old female OKC-HET^B^ rats.

## DISCUSSION

Over the past two decades, investigators have studied how aging impacts the gut microbiome. Laboratory rodents have emerged as an excellent model to study microbiome changes with age because variables that can affect the microbiome composition in humans, such as diet and environment [12], can be controlled over the lifespan of the animal. One of the limitations in previous rodent studies is that they have used inbred mouse strains [15–18, 24]. Because the genome of the host has been shown to affect the gut [34–36], it is unknown how well microbiome changes with age in one inbred mouse strain will translate to another inbred strain or to a genetically diverse population, such as that encountered by investigators in studying humans. This problem is exacerbated because all aging studies in mice have used only one inbred strain, C57BL/6 mice. While several studies have used outbred rat strains, e.g., Wistar or Sprague Dawley rats [19–21], the genetic variation is limited in these outbred rats [46] and can vary considerably from one commercial source to another [47]. To overcome these limitations, we used a genetically heterogenous rodent model, the OKC-HET^B/W^ rat, which was generated to maximize genetic heterozygosity [48]. This breeding strategy not only allows us (or other investigators) to generate similar genetically heterozygous rats at any time; but also allows us to generate two strains of rats that differ in their mitochondrial genomes, e.g., mitochondria from either BN (OKC-HET^B^) or WKY (OKC-HET^W^) rats. Several reports (Hirose et al., Kunstner et al., and Yardeni et al.) indicate that mt-haplotype can alter the microbiome of young C57BL/6 mice [37–39]. Thus, the OKC-HET^B/W^ rat gives us the first opportunity to determine if the mt-haplotype has an impact on the age-related changes in the microbiome in a genetically heterozygous animal model. Another advantage of using the OKC-HET^B/W^ rat model is that rats are more similar to humans in many respects than laboratory mice used in research, e.g., insulin sensitivity, muscle biology, cognition testing, social behavior, and sex differences in longevity [49, 50]. In addition, most laboratory strains of mice die primarily from cancer, while rats also die from non-neoplastic leasions such as heart and reneal diseases [49], which could impact the microbiome in old animals. Therefore, the data generated from the OKC-HET^B/W^ rats are likely to be more translatable to humans.

To determine how the gut microbiome changed with age, we compared the abundance of microbial species in adult (9 months) and old (26 months) male and female OCK-HET^B/W^ rats. A total of 11 microbial species changed significantly with age: five species in male rats and nine species in female rats. Only three microbes changed significantly with age in the same direction in both male and female rats. On the other hand, six species changed with age only in females. Thus, we found that the sex of the host played an important role in shaping the microbiome changes that occur with age in the OKC-HET^B/W^ rat. Currently, there are only two studies that have compared the effect of sex on the age-related changes in the microbiome: one in rats [51] and one in humans [52]. Lee et al. studied the effect of feeding a high-fat diet to young rats (6 weeks) and old (2 years) inbred F344 rats [51]. While they found that feeding a high-fat diet significantly altered the microbiome of young and old male and female rats, they did not find any significant age-related change in relative abundance of microbes in either male or female chow fed F344 rats. The limited number of rats studied (especially the old rats) and/or the collection of fecal material after defecation, which can impact the microbiome composition [53], could explain the differences in our findings. Takagi et al. [52] studied the gut microbiome in healthy Japanese subjects ranging from 20 to 89 years of age. There were no significant changes in the microbiome with age observed at any level of taxonomy in either males or females separately. Interestingly, significant differences were observed in the microbiome between male and female participants at each age group studied. Fourteen genera were increased specifically in males compared to females, and 11 genera were significantly increased in female compared to male participants. However, these differences could be due to sex hormones [54–56] or differences in behavior (e.g., smoking, alcohol consumption, activity) [57] or diet [58]. Our data clearly demonstrate for the first time that sex of the host had a major impact on the age-related changes in the gut microbiome and emphasizes the importance of studying both males and females when investigating the impact of aging on the microbiome.

The OKC-HET^B/W^ rat model allowed us to study the effect of the mt-haplotype on the age-related changes in the gut microbiome for the first time. Previous studies have reported that the mitochondrial genome of the host could affect the microbiome of young, inbred mice where male and female mice were grouped together for analysis [37–39]. However, these studies are limited because the mitochondrial genomes compared differed by only two to five nucleotides, which is a result of the traditional inbred strains of laboratory mice originating from a single female *Mus musculus domesticus* mouse [59]. In addition, the different mitochondrial genomes were on the same inbred background (C57BL/6). Because the B- and W-haplotypes are on a genetically heterogenous background, any differences we observe in the OKC-HET^B^ and OKC-HET^W^ rats are robust, i.e., changes occur on multiple nuclear genotypes and are therefore more likely to occur in humans. We observed that most of the microbial species that changed significantly with age occurred in one but not both mt-haplotypes. For example, nine out of 11 microbes that changed with age were mt-haplotype specific and occurred in one sex and not the other. Previous work in humans have associated increases in abundance of the genera *Bifidobacterium* and* Akkermansia* to Alzheimer’s [9, 10] and Parkinson’s [11] diseases respectively. In the female OKC-HET rats, species in the genera *Bifidobacterium* and *Akkermansia* were changed with age in a mt-haplotype specific manner. Although the previous studies did not show any association between the mt-haplotype and microbial species, they reported significant associations at the genus [37, 38] and family [39]. We also found mt-haplotype differences at the level of the genus and order. For example, the genus *Lachnospira* was increased in adult male OKC-HET^W^ compared to adult male OKC-HET^B^ rats. The family Lachnospiraceae (containing genus *Lachnospira*) was reported to differ with mt-haplotypes in C75BL/6 mice [37, 39] and was negatively correlated with reactive oxygen species production [39]. We found the genus *Bilophila* was significantly increased in adult female OKC-HET^W^ rats compared to adult female OKC-HET^B^ rats. The family Desulfovibrionaceae, containing the genus *Bilophila*, was reported to be significantly increased in C57BL/6J mice with the FVB/NJ mouse mt-haplotype compared to their conplastic pairs containing C57BL/6 nuclear DNA and NZB/BlnJ mt-haplotype [37]. Finally, we observed that the order Verrucomicrobiales was significantly increased in old female OKC-HET^W^ compared to old female OKC-HET^B^. These observed differences when comparing mt-haplotypes show that mt-haplotype can influence microbiome composition in a genetically heterogenous animal model.

To determine how the changes in the microbiome might impact the aging host, we measured the SCFAs. SCFAs are produced from fermentation of indigestible fiber from commensal bacteria in the gut and can regulate metabolic pathways in the host [42]. We observed a trend for an age-related increase in total fecal SCFAs in female rats with female OKC-HET^W^ rats having a significant increase in total SCFAs with age. This change with age can be attributed to increased acetic acid in old female OKC-HET^B^ and OKC-HET^W^ rats and increased propionic acid in old female OKC-HET^W^ rats. Previous studies in humans have reported that fecal SCFAs decrease with age in both male and female human participants [60] and when male and female participants are combined [61]. Additionally, Lee et al. [51] found that cecal SCFAs decreased with age in male F344 rats. However, fecal SCFAs were observed to increase in obese compared to lean individuals [62] and was positively correlated with risk factors associated with metabolic syndrome in women such as increased adiposity [63]. Therefore, we compared the level of total fecal SCFAs to the fat mass of the individual female rats. As shown in Supplementary Figure 5, we observed a positive correlation between total fecal SCFAs and subcutaneous fat mass that tended towards significance (*p* = 0.07, r = 0.41) in female rats. When separating female OKC-HET^W^ rats, there was a significant positive correlation (*p* = 0.04, r = 0.65). The changes in SCFAs in feces could arise from an increase in microbes that produce SCFAs [64, 65] or from decreased absorption of SCFAs into plasma [66]. Because most of the bacteria known to produce SCFAs, e.g., Lactobacillaceae and Ruminococcaceae families [42], decreased with age in our rats (Figure 3), the increased SCFAs we observed in feces of the old female rats most likely arises from a decrease in the absorption of the SCFAs into the plasma, which could arise from the age-related decline in intestinal absorption that has been reported in rats [67], mice [68], and humans [69].

Tryptophan metabolites are one of the best examples of the metabolic cross talk between the gut microbiome and host because these metabolites can influence the host’s health and disease processes [43, 70]. Tryptophan is metabolized by microbes into indoles, which can modulate pathways producing either serotonin or kynurenine from tryptophan. The production of serotonin and kynurenine are primarily produced by the host’s endochromaffin cells [71, 72] and liver [73, 74], respectively. However, indole and its derivatives are produced specifically by gut microbiota [43]. We observed major differences in plasma metabolites of tryptophan by sex. The total level of plasma indoles tended to be lower in adult and old female OKC-HET^W^ rats compared to OKC-HET^B^ rats (Supplementary Figure 3D). Four tryptophan metabolites changed with age in female rats and only one in males (Figure 6). The plasma levels of kynurenine showed the greatest change with age in females. Although the mechanism through which the microbiome impacts liver kynurenine production is unclear, studies using germ-free mice have shown that plasma levels of kynurenine are significantly reduced in the absence of the microbiome [73], which is increased after recolonization [75]. Plasma kynurenine decreased significantly with age in both mt-haplotypes in female rats. Although kynurenine levels did not change significantly with age in male rats, they were marginally decreased in male OKC-HET^B^ compared to male OKC-HET^W^ rats. A study in male inbred mice reported age-related increases in serum kynurenine levels between 3 and 28 months of age [18]. However, Comai et al. [76] reported an age-related decrease in enzymatic activity of liver indoleamine 2,3-dioxygenase (IDO) in Sprague-Dawley rats. IDO is the rate-limiting step in tryptophan metabolism to kynurenine and is modulated by the gut microbiome [43], which could agree with our observation that kynurenine levels decrease with age in rats. In a review. Bakker et al. reported that 50% of the human studies, which measured plasma kynurenine with age, found increased plasma kynurenine levels [77]. Interestingly, for all the tryptophan metabolites that changed with age, we observed mt-haplotype differences in adult rats. For example, kynurenine and hydroxykynurenine levels were reduced in female OKC-HET^W^ rats while in male rats, kynurenine was increased in male OKC-HET^W^ rats. Thus, mt-haplotype appears to play a role in tryptophan metabolism, which could occur via modulation of gut microbiome composition.

The microbiome also plays an important role in the bile acid pool size and composition [78]. Primary bile acids (PBAs) produced in the liver are secreted into the gastrointestinal tract of rats. Because primary conjugated bile acids are detergents and acidic, they can affect the gut microbe diversity and composition [79, 80]. While most of the PBAs are reabsorbed in the terminal ileum, the gut microbiota can deconjugate and metabolize them into secondary bile acids, which are absorbed in the terminal ileum and colon [78]. These secondary bile acids produced by the microbiome play an important role in bile acid homeostasis of the host, and these changes in secondary bile acids can contribute to conditions like non-alcoholic fatty liver disease, inflammatory bowel disease, and cholesterol disorders [80–82]. We observed significant changes in the plasma levels of only PBAs with age and mt-haplotype. In contrast to tryptophan metabolites, the age-related changes in plasma levels of bile acids were greatest in male rats, with five metabolites changing with age or mt-haplotype compared with two metabolites in female rats. In general, there was an age-related increase in plasma bile acids in male but not female rats (Supplementary Figure 4). However, total PBAs were marginally significance with age only in male OKC-HET^B^ rats. These changes can be attributed to increases in the plasma levels of TUDCA, TCDA, GCA, and GUDCA in OKC-HET^B^ male rats. Bile acid secretion from the liver decreases with age in male and female humans as measured from the biliary duct [83] and is unaffected in aged male Wistar and Sprague Dawley rats [84]. In humans, plasma bile acids decrease with age in males while female plasma bile acid profile is largely unchanged [85]. However, our data support previous work in male rats suggesting increased plasma primary bile acids with age. For example, an increase in plasma bile acids was reported in male Wistar-Imamichi rats between three and 11 months of age [86], and taurine-conjugated bile acids were increased in bile collected from the bile duct between six and 15, months of age in male Sprague-Dawley rats [87]. We also observed that mt-haplotype had an impact on the plasma levels of the primary bile acids. Except for LCT, which decreased with age in both the B- and W-haplotypes in female rats, all the changes in bile acid metabolites were mt-haplotype specific. Particularly striking was the age-related increase in TUDCA, TCDCA, GCA, and GUDCA, which was observed only in male OKC-HET^B^ rats. We did not observe any differences in secondary bile acids when comparing age or mt-haplotype in male and female OKC-HET^B/W^ rats. Thus, age and mt-haplotype appear to play a greater role in the host’s ability to produce PBA with age than the plasma secondary bile acids produced by the microbiome. PBAs are known to influence gut microbiome composition because of their antimicrobial properties and by increasing the acidic environment of the colon [79, 80]. Therefore, we were interested in determining if any microbes changed specifically with age only in male OKC-HET^B^ rats that might be associated with the increase in plasma levels of TUDCA, TCDCA, GCA, and GUDCA. A significant increase in the abundance of *L. reuteri* was observed in old male OKC-HET^B^ rats that was associated with the age-related increase with PBAs in plasma of the male OKC-HET^B^ rats. Because *L. reuteri* is a lactobacillus species that is resistant to an acidic environment [79], the age-related increase in *L. reuteri* in the male OKC-HET^B^ rats might have arisen from increased PBAs in the colon that came from the increased levels of plasma PBAs.

In conclusion, we used a genetically heterogenous rat model to study how the gut microbiome changes with age that should be more translatable to humans than previous studies with inbred mice or outbred rats, which have limited genetic diversity. We found the age-related changes in the microbiome differed greatly between male and female rats, demonstrating the importance of studying both males and females when evaluating the impact of age on the microbiome. Importantly, we found that the mt-haplotype of the rats played an important role in how aging altered the microbiome. Although previous studies have shown the mitochondrial genome can affect the microbiome of young, inbred mice [37–39], it was not clear from these studies if the effect of mt-haplotype would translate to other genetic backgrounds or differ with sex. Our data show for the first time that mt-haplotype differences are robust enough to impact the microbiome on a genetic heterogenous background. In addition, we found that the effect of the mt-haplotype was sex dependent, i.e., the impact of the mt-haplotype on the age-related changes in the microbiome almost always occurred in one sex and not the other. Because the microbiome has been shown to impact a host’s metabolic health [3], immunity [4], cardiovascular health [5], and cognitive function [6, 7], these age-related changes in the microbiome could play a role in the increased occurance of disease and pathology seen in older individuals.

Recent data suggest a bidirectional interaction between the gut microbiome and mitochondria [73, 88, 89]. For example, an early study by Han et al. (2017) with *C. elegans* showed that several *E. coli* mutants promoted longevity through the secretion of colonic acid, which regulated mitochondrial dynamics and the unfolded protein response in the host’s cells. In addition, two species of *Lactobacillus* [90] and postbiotics from *Lacticaseibacillus casei* [91] were reported to alter mitochondrial function in the liver of rats. On the other hand, the deficiency of the mitochondrial protein (methylation-controlled J protein) in mice was shown to have profound effect on the microbiome [88, 92]. Thus, the question emerges as to how the microbiome and host mitochondria communicate. In a review, Zhang et al. (2022) proposed that SCFAs produced by the microbiome could be modulators of mitochondria function in the intestinal epithelium [89]. Interestingly, we showed that fecal SCFAs were increased with age in female rats. Yardeni et al. (2019) showed that differences in the mitochondrial redox status and ROS production that occurred in mice with different mitochondrial genomes were associated with modifications in the gut microbiome [39]. They proposed that changes in redox status might impact metabolites produced by the host cells that were then secreted into the gut. They also showed that expressing catalase in the mitochondria of the host had the single greatest impact on the gut microbiome, suggesting hydrogen peroxide produced by the host might act directly on the microbes in the gut. They also proposed that the release of mtDNA from the mitochondria in stressed intestinal cells could activate the cGAS-Sting pathway, resulting in an inflammatory response that could in turn affect the gut microbiome. Thus, to understand the impact of mt-haplotype on the gut microbiome, future studies should focus on how mitochondrial function differs in the cells in the intestine of OKC-HET^B^ and OKC-HET^W^ rats as they age. Our preliminary data show that feeding a high-fat diet affected mitochondrial function in skeletal muscle differently in the B- and W-haplotypes [48].

## MATERIALS AND METHODS

### Animals

The OKC-HET^B/W^ rats with two different mitochondrial haplotypes were generated by breeding four inbred strains of rats ((Brown Norway (BN), Fischer 344 (F344), Wistar Kyoto (WKY), and Lewis (LEW) rats) obtained from Charles River as previously described [48]. Briefly, BN/F344 F1 rats were generated by crossing female BN rats to male F344 rats and WKY/LEW F1 rats were generated by crossing female WKY to male LEW rats (WKY/LEW). OKC-HET^B^ rats were generated by crossing female BN/F344 to male WKY/LEW rats and the OKC-HET^W^ were generated by crossing female WKY/LEW to male BN/F344 rats. Due to this selective breeding and taking advantage of the maternal inheritance of mitochondrial DNA, all rats have similar nuclear heterogeneity while containing two different mitochondrial haplotypes. The OKC-HET^B^ rats contain mitochondria from the BN rats, and the OKC-HET^W^ rats contain mitochondria from the WKY rats, which differ by 94 nucleotides [48]. These rats were bred and maintained in the Oklahoma City VA Medical Center animal facilities in specific pathogen free conditions. They were fed *ad libitum* on chow diet (Picolab Rodent Diet 5053, LabDiet, St. Louis, MO, USA). Male and female OKC-HET^B^ and OKC-HET^W^ rats were studied at 9- (adult) and 26- (old) months of age. Feces and plasma were collected from adult male OKC-HET^B^ (*n* = 6), adult male OKC-HET^W^ (*n* = 6), adult female OKC-HET^B^ (*n* = 9), adult female OKC-HET^W^ (*n* = 9), old male OKC-HET^B^ (*n* = 10), old male OKC-HET^W^ (*n* = 6), old female OKC-HET^B^ (*n* = 7), and old female OKC-HET^W^ (*n* = 8) rats. Rats were fasted for 16 hours prior to termination and whole blood was collected in EDTA coated tubes by cardiac puncture, and plasma was separated from whole blood (1000 × g for 10 minutes), flash frozen, and stored at −80°C. The colon was separated from the anus and feces were collected directly from the colon, immediately flash frozen in liquid nitrogen, and stored at −80°C. All procedures were approved by the Institutional Animal Care and Use Committee at the Oklahoma City Veterans Affairs Health Care System (Protocol Number: 1640635/2108-002).

### Microbiome analysis

#### DNA extraction

DNA was isolated from colon fecal samples using the ZymoBIOMICS DNA miniprep Kit (D4300, ZYMO RESEARCH, Orange, CA, USA) as specified by the manufacturer. A NanoDrop Lite Spectrophotometer (ThermoFisher Scientific) was used to determine quantity and quality of isolated DNA.

#### 16S rRNA sequencing

Library construction and 16S rRNA sequencing were performed on the isolated fecal DNA by the OUHS Institutional Research Core Facility (IRCF). Data were generated using Illumina MiSeq libraries prepared using MiSeq Reagent Kit V3-V4. Data analysis was provided by the OK-INBRE Data Science Core. Sequences were processed and analyzed using QIIME2 v2022.11 [93]. Standard data clean-up was performed using Cutadapt [94] Sequences were grouped into amplicon sequence variants (ASVs) using the DADA2 QIIME2 plugin [95]. MAFFT was used to align the ASVs and FastTree was used to create a rooted phylogenetic tree [96, 97]. Rarefaction curves showed that all samples reached asymptote indicating the sequencing depth used was sufficient. A QIIME2 naïve Bayesian classifier trained on sequences from the V3-V4 region of Greengenes v13_8 99% OTUs was used to assign a taxonomic profile to each ASV [98]. Microbial abundance tables were generated to the species level.

### Analysis of plasma metabolites

#### Metabolite extraction

Extraction of metabolites from rat plasma was adapted from a previous study [90]. Metabolites from 70 µL of plasma were extracted in 400 µL cold methanol/acetonitrile (1:1, v/v) and homogenized in a Precellys 24 Touch Homogenizer (Bertin technologies, Montigny-le-Bretonneux, France) twice for 20 seconds. The samples were then incubated for two hours at −20°C to precipitate protein. Samples were centrifuged at 4°C for 15 minutes at 13,000 × g, and 400 µL of supernatant was collected. The supernatant was centrifuged again at 4°C for 15 minutes at 13,000 × g. Supernatant was collected and evaporated to dryness in a vacuum concentrator for two hours. The dry extracts were reconstituted in 150 µL of acetonitrile/DI water (1:1, v/v) and then vortexed for 30 seconds to dissolve the dried metabolite pellet. The samples were centrifuged for the last time at 4°C for 10 minutes at 13,000 × g. A quality control (QC) pooled sample was prepared by combining 5 µL of each sample supernatant. The QC is a “mean” profile representing all metabolites encountered in the analysis. The sample supernatants and QC were stored at −80°C until they were analyzed using liquid chromatography with tandem mass spectrometry (LC-MS/MS).

#### LC-MS/MS-based metabolomics analysis

Samples were spiked just before analysis with 3 µL of isotope labeled metabolite Mix 1 QReSS Kit (Cambridge Isotope Labs) to account for potential instrument performance variation throughout the analysis. Supplementary Figure 6. shows that our range of variation between the expected m/z and measured m/z was less than 0.05 ppm. Liquid chromatography was performed using a Sciex ExionLC AD ultra-high-performance liquid chromatograph (UPHLC) system. Volumes of 2 µL were injected into a 2.1 × 150 mm, 2 µL Intersil Ph-3 HPLC column (GL Sciences, Torrance, CA, USA). The autosampler and column over temperature were held at 15°C and 40°C, respectively. A binary elution system of (A) LC-MS grade water + 0.1 % formic acid and (B) methanol + 0.1% formic acid was utilized to achieve separation using a flow rate of 0.3 mL/min in a 23 min gradient. Pooled QC and blanks were injected between every eight samples, and samples were fully randomized prior to injection. Mass spectrometry analysis was conducted with a Sciex ZenoTOF 7600 (Framingham, MA, USA) in both positive and negative electrospray ionization modes, utilizing the information-dependent acquisition (IDA) mode.

#### LC-MS/MS data processing

Raw data was imported into MS Dial v5 (RIKEN Center, Yokohama City, Kanagawa, Japan) [99] for feature detection, peak alignment, and peak integration. Metabolites were confirmed using MS, MS/MS fragmentation using publicly available libraries for LC-MS/MS from the MassBank of North America (MoNA), and an in-house curated IROA library (Ann Arbor, MI, USA). Data was acquired in both electrospray ionization (ESI) positive and negative modes. First, features were removed from the dataset if their peak height was not at least 5-fold higher than that found in blank samples. Features without MS/MS peaks, or known fragmentation signatures, were removed. Metabolites associated with microbial deconjugation of bile acids (primary and secondary bile acids) and tryptophan metabolism (serotonin, kynurenine, indoles) were selected from the metabolome for further data analysis [43, 80, 100].

### Targeted metabolomics for fecal short-chain fatty acids (SCFAs)

Colonic feces (200 mg) of five randomly selected rats per group were sent to Metabolon (Metabolon, Inc., Morrisville, NC, USA) where SCFAs were quantified with a targeted metabolomic LC-MS/MS (Agilent 1290 UHPLC/Sciex QTrap 5500) panel of eight total metabolites. All eight metabolites were detected in our samples.

### Statistical analysis

Statistical analysis was completed using MicrobiomeAnalyst 2.0 [101], MetaboAnalyst 6.0 [102], and GraphPad v10 (Dotmatics, Boston, MA, USA). Statistical analysis of male and female rats was completed separately. Comparisons of age (adult vs. old) were performed in rats of the same sex and mt-haplotype. Comparisons of mt-haplotype were performed on rats of the same age and sex but different mt-haplotypes (e.g. adult male OKC-HET^B^ vs. adult male OKC-HET^W^). ANOVA with Tukey’s post-hoc analysis was used to determine differences in body weight, subcutaneous fat weight, and gonadal fat weight. *T*-test was used to compare fat weights in old female OKC-HET^B/W^ rats.

MicrobiomeAnalyst 2.0 was used to determine differences in Chao1 and Shannon alpha-diversity using Mann-Whitney/Krushkal-Wallis FDR <0.05 cutoff for significance. Differences in beta-diversity were determined using Jensen-Shannon Divergence and PERMANOVA FDR <0.05 to determine significance and were represented by principal coordinate analysis (PCoA) plots. Differences in abundance of individual microbes at different levels of taxa were determined using Mann-Whitney/Kruskal-Wallis (MK) FDR <0.05 and Linear Modeling (LM) from MaAsLin2 [51] integrated in MicrobiomeAnalyst 2.0 after normalizing data using relative log expression. For ease of understanding, all significant differences are represented as relative abundance (%). SparCC [103] and SECOM (Pearson1) [104] algorithms embedded in MicrobiomeAnalyst 2.0 were used to determine linear correlation relationships between microbial species to other microbial species in rats of the same age, sex, and mt-haplotype. SparCC was run with 100 permutations. A *p*-value < 0.05 and correlation threshold of 0.3 was considered significant for both SparCC and SECOM (Pearson1) analyses.

Statistical analysis of metabolite content was performed in GraphPad v10. Heat maps were created using MetaboAnalyst 6.0. Comparisons were completed in male and female rats separately. Fisher’s LSD with FDR <0.05 was used to determine significance. Additionally, differences in metabolites were considered to be marginally significant when Fisher’s LSD <0.5, and FDR >0.05. This was done to bring attention to metabolites that could be physiologically important though they did not meet the arbitrary significance value of *p* = 0.05. All variables were checked for normality and non-parametric tests were utilized if the test for normality was significant (i.e., the data were not normal).

## Supplementary Materials

Supplementary Figures
